# Faculty Perspectives on AI Integration in Anatomy Education in the United Arab Emirates: Cross-Sectional Survey

**DOI:** 10.2196/87418

**Published:** 2026-04-21

**Authors:** Prince Last Mudenda Zilundu, Pedzisai Mazengenya, Jayaraj Kodangattil Narayanan, LiHua Zhou

**Affiliations:** 1 Basic Medical Sciences Ajman University Ajman United Arab Emirates; 2 Basic Medical and Dental Sciences Ajman University Ajman United Arab Emirates; 3 Sun Yat-sen University Shenzhen China

**Keywords:** artificial intelligence, anatomy education, faculty perspectives, SWOT, strengths, weaknesses, opportunities, and threats, medical education, United Arab Emirates, professional development, policy, assessment, extended reality, 3D visualization

## Abstract

**Background:**

Artificial intelligence (AI) is reshaping medical and health professions education; yet, adoption in anatomy remains uneven and often ad hoc. Anatomy’s spatial and visualization demands make it a compelling domain for AI, but discipline-specific opportunities and risks are not well characterized in the United Arab Emirates.

**Objective:**

This study examines United Arab Emirates anatomy educators’ AI use, attitudes, perceived barriers and enablers, and strategic perspectives on AI integration using a design informed by the Unified Theory of Acceptance and Use of Technology 2 (UTAUT2).

**Methods:**

A cross-sectional survey of anatomy faculty at United Arab Emirates medical and health sciences colleges used 5-point Likert items to assess educational technology proficiency, AI use patterns, AI attitudes, perceived barriers and facilitators, and professional development needs. Quantitative data were summarized descriptively and explored with nonparametric tests. Open-ended strengths, weaknesses, opportunities, and threats questions were analyzed using reflexive thematic analysis, organized within the strengths, weaknesses, opportunities, and threats framework, and interpreted through UTAUT2 constructs. Quantitative and qualitative strands were integrated at interpretation through triangulation.

**Results:**

In total, 30 anatomy faculty participated. Self-rated educational technology proficiency was high (mean 3.73 out of 5, SD 1.01), and overall attitudes toward AI in anatomy education were positive (mean 4.23, SD 0.73), with strong interest in AI-focused professional development (mean 4.50, SD 0.73). Most respondents reported using generative AI tools, predominantly ChatGPT, for content creation, quiz and examination item generation, summarization of complex material, and, to a lesser extent, visualization and workflow streamlining. Capacity-related barriers predominated: time and workload pressures (mean 3.27, SD 1.17) and training gaps (mean 3.13, SD 1.22) were rated as moderate obstacles, whereas budget or resource limitations (mean 2.63, SD 1.19) and academic integrity concerns (mean 2.80, SD 1.10) were minor obstacles. Student interest (mean 4.23, SD 0.86) and institutional encouragement (mean 4.00, SD 1.14) emerged as strong facilitators, with no statistically detectable differences by academic rank, age, or years of experience in this small, underpowered sample. Qualitatively, themes highlighted strong institutional support and digital readiness as strengths; training needs, workload, and policy gaps as weaknesses; visualization, personalization, and efficiency as opportunities; and overreliance, ethical risks, and erosion of hands-on anatomy pedagogy as threats. UTAUT2 interpretation indicated high performance expectancy and social influence (student and institutional support) but reduced effort expectancy and facilitating conditions due to time, training, and governance constraints, collectively tempering behavioral intention.

**Conclusions:**

In this exploratory sample, United Arab Emirates anatomy educators were broadly receptive to generative AI and already experimenting and valuing the benefits for 3D visualization, adaptive practice, and feedback. However, workload, limited training, and unclear governance (disclosure, assessment integrity, and cadaveric or patient images) constrain uptake, underscoring the need for protected time, workflow-aligned training, and discipline-specific policies to enable sustainable, ethical integration.

## Introduction

Artificial intelligence (AI) is rapidly transforming medical and health professions education. For instance, intelligent tutoring systems and adaptive learning platforms can diagnose knowledge gaps and deliver tailored remediation, supporting self-directed and mastery-based learning [1]. AI-driven simulation is enhancing experiential training through virtual and augmented reality environments as well as synthetic patient encounters, allowing learners to practice decision-making safely and repeatedly [2]. Assessment has similarly been reshaped by automated scoring, pattern recognition, and feedback algorithms that provide rapid, personalized performance insights [3,4]. These innovations extend beyond content delivery to student engagement, where conversational agents, game-based adaptive systems, and learning analytics have improved motivation and interactivity [5-7]. Yet, the educational impact of these tools ultimately depends on educators’ willingness to adopt and integrate them into curricula.

Although much popular attention has focused on large generative models such as ChatGPT, the AI landscape in medical education is broader. Natural language processing, image analysis, and intelligent search underpin a growing range of educational tools from anatomy image annotation and question generation to personalized study schedules [8]. Global surveys show that while students adopt AI quickly, faculty uptake is uneven; educators express both interest and uncertainty about safe and pedagogically sound integration [9-11]. Recent scoping reviews and analyses emphasize that generative artificial intelligence (GenAI) adoption in formal medical curricula remains early and often unstructured [12-14]. Considering these patterns, this study contributes discipline-specific, United Arab Emirates–based evidence by detailing how anatomy educators use as well as perceive AI’s benefits, risks, and practical enablers and by indicating where structured support is most needed next*.*

Anatomy, a fundamental subject in medical and health professions education, is particularly well-suited for AI-supported innovation. The subject’s large knowledge base, reliance on spatial reasoning, and traditional use of cadaveric resources create long-standing challenges for visualization and cognitive load [15]. AI can augment 3D understanding through advanced segmentation and labeling of images, adaptive quizzing, and mixed-reality simulations [16,17]. Early work combining AI with augmented and virtual reality shows promise for improving anatomical comprehension and retention [18]. However, these opportunities bring discipline-specific questions: how do anatomy educators view AI’s role or adoption alongside dissection and prosection, and what support do they need to use it responsibly?

The Unified Theory of Acceptance and Use of Technology (UTAUT) and its extension Unified Theory of Acceptance and Use of Technology 2 (UTAUT2) offer a robust framework to examine this adoption, highlighting how performance expectancy (improving teaching), effort expectancy (ease of use), social influence, and facilitating conditions (support from peers or institutions), as well as hedonic motivation and habits (personal enjoyment and prior experience), shape technology-related behavioral intentions in medical education settings [19,20]. Recent studies applying the UTAUT framework to faculty adoption of AI in higher education have shown that UTAUT constructs significantly influence educators’ behavioral intentions to integrate AI into teaching and learning [21]. Applying this framework allows for systematic exploration of anatomy educators’ perceptions, intentions, and contextual barriers to AI use in medical education. However, technology adoption theories [22], while theoretically robust, may not fully capture the discipline-specific, institutional, and context-grounded realities that emerge from educator experiences in specialized fields such as anatomy, where pedagogical traditions (cadaveric dissection, hands-on learning) are deeply embedded [23,24]. Consequently, this study triangulates the deductive, theory-driven UTAUT2 framework with an inductive strengths, weaknesses, opportunities, and threats (SWOT) analysis. This mixed methods approach provides both theoretical coherence, through UTAUT2’s operationalization of adoption constructs, and practical, context-specific insights that illuminate the particular barriers and enablers relevant to anatomy education. The SWOT analysis captures anatomy educators’ lived experiences, institutional constraints, and aspirations without forcing findings into predetermined theoretical categories, thereby enriching the applicability of adoption theory to a specialized educational context [25]. In the United Arab Emirates, where institutions are advancing national digital transformation agendas [26], examining faculty perspectives on AI integration is timely and essential to inform policy, professional development, and curriculum innovation.

Globally, faculty report persistent concerns about AI-related academic integrity, superficial learning, and uneven, early-stage uptake, underscoring the need for explicit institutional frameworks [27-29]. In anatomy education, these issues are amplified by governance over cadaveric and patient images as well as by threats to assessment validity [13]. In the Middle East and North Africa, the evidence base remains emergent and predominantly student-focused rather than faculty or discipline-specific [30,31]. For example, in Saudi Arabia, the few faculty-oriented studies report moderate awareness but uneven competence and a need for structured training, alongside concerns about policy inconsistencies, academic integrity, and data governance issues [32-34]. Published United Arab Emirates data are particularly scarce and largely student-focused; to our knowledge, no peer-reviewed study has yet mapped how anatomy educators in the United Arab Emirates perceive AI in their teaching and assessment contexts [31,35].

This study sought to bridge this evidence gap by exploring the perspectives of anatomy educators in the United Arab Emirates regarding AI integration in medical and health professions education. Specifically, this study aimed (1) to examine anatomy educators’ use and perceived benefits or risks of AI in teaching and assessment; (2) to assess their readiness and willingness to adopt AI-supported pedagogical tools in their discipline; and (3) to identify the availability and adequacy of institutional support, policy frameworks, and professional development needed to enable responsible and pedagogically sound AI integration. Understanding these views is essential to inform responsible, discipline-appropriate AI adoption in the region and to align global innovations with local educational realities.

## Methods

### Study Design and Reporting

This study adopted a cross-sectional design integrating quantitative survey data and qualitative thematic analysis to explore anatomy educators’ use patterns and perspectives on AI integration in teaching and assessment. Reporting follows STROBE (Strengthening the Reporting of Observational Studies in Epidemiology) for observational cross-sectional studies and relevant CHERRIES (Checklist for Reporting Results of Internet E-Surveys) items for web surveys; qualitative analysis is described in line with SRQR (Standards for Reporting Qualitative Research) and reflexive thematic analysis guidance. Survey prompts and the full instrument are provided in Multimedia Appendix 1. Checklists (STROBE, CHERRIES, and SRQR) are present in Multimedia Appendix 2.

### Setting and Recruitment

Data were collected at the 4th Annual Anatomy and Cellular Biology Conference (Dubai Medical College; May 10-11, 2025), an annual national forum drawing anatomy faculty (AF) from all United Arab Emirates institutions. The conference served as an ideal recruitment setting due to its disciplinary focus, diverse institutional representation, and concentration of target participants in a single venue. Conference organizers announced the study during the opening plenary, directing attendees to the printed QR codes at the venue. Research staff (in acknowledgements) provided a brief orientation and offered 2 completion options: email and on-site (via QR codes). Recruitment was extended 2 weeks (May 12-25) via institutional faculty email lists, United Arab Emirates Anatomy Society WhatsApp group (41 members), and snowball referrals. All invitations emphasized voluntary participation, anonymity, independent completion, and absence of institutional coercion. Surveys were delivered via Microsoft Forms.

### Participants and Eligibility

Eligible participants were faculty actively teaching anatomy or closely related subdisciplines (eg, gross anatomy, histology, neuroanatomy, embryology, or anatomy and physiology) in undergraduate medical, dental, or allied-health programs in the United Arab Emirates. Inclusion required current teaching involvement and electronic consent. At the time of the study, anatomy courses were offered across 16 colleges of medicine, pharmacy, and health sciences in the country.

### Quantitative Data Collection

The structured questionnaire was derived from contemporary literature on AI in health professions education and adapted to faculty contexts [7,12,32,36]. Item development was also informed by the UTAUT2 [19,20], which conceptualizes 6 key determinants of technology adoption: performance expectancy, effort expectancy, social influence, facilitating conditions, hedonic motivation, and habit. These constructs guided the formulation of items assessing faculty perceptions, institutional support, and behavioral intentions toward AI integration.

The quantitative component comprised 5-point Likert-type items (1=strongly disagree to 5=strongly agree), multiple-choice questions, and closed ended checklists organized across four domains: (1) demographics and teaching background, (2) familiarity with educational technology and current use of AI tools, (3) perceived benefits and challenges (including ethics, integrity, privacy, and governance), and (4) professional development interest and future adoption. Each quantitative item explicitly operationalized 1 or more UTAUT2 constructs: performance expectancy (benefit-related items), effort expectancy (ease-of-use items), social influence (peer and leadership support items), facilitating conditions (enabler items), hedonic motivation (enjoyment and satisfaction items), habit (prior experience items), and behavioral intention (stated willingness to adopt). This quantitative strand generated the primary and secondary outcomes described in the Outcomes and Measures section.

### Qualitative Data Collection

To capture more nuanced faculty perspectives that could not be fully represented through closed-ended items, the survey incorporated qualitatively oriented open-ended questions organized using the SWOT framework. These SWOT prompts functioned as the primary mechanism for qualitative data collection, inviting free-text narrative responses to elicit detailed, context-rich accounts of faculty experiences and views. The Strengths domain asked educators to identify and describe perceived advantages of AI integration, recount or describe successful experiences, and provide examples of improved teaching or student engagement. The Weaknesses domain invited participants to reflect on and clearly describe observed challenges (technological, pedagogical, institutional, or other), the limitations encountered, and any documented effects on teaching practices or student outcomes. The Opportunities domain explored future applications, novel teaching and research areas, and potential contributions to faculty development and curriculum innovation. The Threats domain elicited concerns about risks, ethical and professional issues, and institutional risk-mitigation strategies. Two additional meta-questions assessed educators’ views on the utility of SWOT analysis for policy development and their recommendations for structured faculty workshops.

Qualitative responses to all SWOT and meta-questions were analyzed thematically using inductive coding to identify recurring themes, negative cases, exemplary quotations, and actionable recommendations. The resulting qualitative themes were then triangulated with the quantitative findings from the structured items to contextualize faculty attitudes, clarify apparent contradictions, and highlight areas of convergence and divergence across data sources. The full mixed methods instrument, including quantitative items and qualitative SWOT prompts, is provided in Multimedia Appendix 1.

### Survey Administration and Data Management

The open survey was hosted on Microsoft Forms. IP addresses or institutional identifiers were not collected. To limit duplicate entries, the landing screen requested 1 response per educator; responses were screened for obvious duplicates (identical role or institution or time-stamp patterns, of which there were none) before analysis. Data were exported to CSV for analysis and stored on an encrypted drive accessible only to the research team. Missing responses were left as missing; denominators are reported per item where applicable.

### Outcomes and Measures

Four primary outcomes were assessed: (1) attitudes toward AI’s role in anatomy education (augment existing practices vs replace traditional methods), (2) perceived benefits of AI (visualization, adaptive learning, assessment efficiency, student engagement, and accessibility), (3) perceived barriers and enablers (technical, pedagogical, ethical or governance, and capacity-related factors), and (4) professional development needs (AI literacy, pedagogy, ethics, technical training, and mentorship). Secondary outcomes included (1) AI familiarity and current use patterns (5-point scale plus yes or no items on use in lectures, assessment, feedback, research, and productivity), (2) differences in perceptions by academic rank (junior, mid-career, and senior), (3) differences by years teaching anatomy (0-5, 6-15, 16-25, and >25 years), (4) differences by age group, and (5) differences by subdiscipline (gross anatomy, neuroanatomy, embryology, and histology). The structured questionnaire used 5-point Likert scales (1=strongly disagree to 5=strongly agree) for all primary outcome items. Multidomain subscales were created for benefits, barriers, and enablers. Composite scores were calculated as means. Open-ended items gathered qualitative elaboration on barriers and professional development needs. Likert items were treated as ordinal. Where applicable, survey items operationalized UTAUT2 constructs: performance expectancy (benefit items), effort expectancy (ease-of-use items), social influence (peer or leadership support), facilitating conditions (enabler items), hedonic motivation (enjoyment), habit (prior experience), and behavioral intention (stated willingness to adopt).

### Quantitative Analysis

Five negatively framed attitude items were reverse-coded (6-original score) to ensure consistent directionality, where higher scores indicate more positive attitudes toward AI in anatomy education across all items. Given the small sample size (N=30) and subgroup imbalance (subgroup n ranges 1-18), all subgroup comparisons are framed as exploratory and descriptive rather than inferential. Likert items and variables failing normality were summarized using nonparametric descriptive statistics (medians and IQRs). To identify preliminary patterns, exploratory group comparisons were conducted using Kruskal-Wallis *H* for multigroup contrasts and Mann-Whitney *U* for 2-group comparisons, with effect sizes (Cliff δ) and 95% CIs reported as aids to interpretation. Where composite scales met normality and internal consistency criteria (α≥.70), Welch ANOVA was used as an alternative. However, readers are cautioned that (1) statistical power is limited for detecting real differences in subgroups; (2) null findings do not imply true group equivalence, only lack of detectable difference; (3) effect size CIs are wide due to small sample size, indicating imprecision; and (4) these exploratory findings should not be interpreted as definitive evidence of group differences and require confirmation in larger samples. No formal type I error control was applied to these exploratory comparisons. All tests were 2-tailed with an α of .05, and analyses were performed in Jamovi (version 2.7.6; The Jamovi Project).

### Qualitative Analysis

Open-ended responses were analyzed using reflexive thematic analysis (Braun and Clarke’s [37] 6 phases): familiarization, inductive coding, theme generation, review, definition or naming, and reporting. Themes were then mapped to the SWOT schema to organize perceptions salient to anatomy teaching and assessment. The coding output was systematically aligned with the 4 SWOT domains, such that each finalized theme and supporting quote were assigned to the quadrant that best represented its perceived valence or strategic implication (eg, internal facilitators under strengths and external challenges under threats). The lead analyst (PLMZ) (an anatomy educator) maintained reflexive memos and an audit trail of coding decisions; peer debriefing with a second anatomist (PM) was used to challenge interpretations and enhance credibility. In line with reflexive thematic analysis, we did not calculate intercoder agreement. Coding was conducted in Microsoft Excel, an approach commonly used in medical-education research, including SWOT-informed curriculum and innovation studies [38]. Representative quotes were selected to illustrate each finalized theme.

### Integration of Quantitative and Qualitative Strands

We used a convergent approach: quantitative and qualitative findings were analyzed in parallel and then integrated at interpretation, using the SWOT map to contextualize descriptive statistics (eg, professional development needs, integrity concerns, and time constraints) within faculty narratives.

### Sample Size and Precision

This was a census-style pragmatic sample of accessible United Arab Emirates anatomy educators at or after an annual national conference. Although no a priori power calculation was feasible, precision is communicated via effect sizes with CIs and transparent reporting of denominators, where necessary. The modest sample (N=30) reflects the practical constraints of recruiting at a single national conference and brief postconference follow-up. Although underpowered for robust subgroup or parametric analyses, it is suitable for descriptive, exploratory work mapping educator perceptions in an understudied United Arab Emirates context. Given the paucity of regional, faculty-focused evidence on AI in anatomy education, a qualitative-led mixed methods approach prioritizes contextual depth over statistical generalizability. Convenience sampling limits representativeness; yet, it yields a diverse mix of anatomy educators across United Arab Emirates institutions, supporting thematic exploration with institutional relevance. Quantitative subgroup comparisons are, therefore, presented as descriptive, not inferential, and the sample size was insufficient for regression or structural equation modeling of UTAUT2 constructs. Further interpretive constraints are detailed in the Study Limitations section.

### Ethical Considerations

The study received ethics approval from Ajman University Institutional Review Board (approval M-F-H-9-May 2025). Participation was voluntary with electronic informed consent. Participants were not compensated for their participation. Data were anonymous and stored securely. As requested by some participants, individual faculty viewpoints were anonymized and not linked to specific institutions; instead, they were aggregated into broader thematic categories.

## Results

### Participant Characteristics

A total of 30 AF members from medical and health sciences institutions across the United Arab Emirates participated in the survey, 3 were excluded due to foreign institutions (Table 1). Most respondents were 40- to 49-year-old assistant or associate professors with ≥11 years of teaching experience, and the majority taught gross anatomy, histology, embryology, and neuroanatomy, with half also teaching anatomy and physiology. Institutions were fully deidentified.

**Table 1:** Demographic and professional characteristics of anatomy faculty respondents who participated in the United Arab Emirates–wide survey on artificial intelligence integration in anatomy education (N=30).

Variable and category		Values, n (%)
**Age group (years)**		
	30-39	6 (20)
	40-49	18 (60)
	50-59	4 (13.3)
	≥60	2 (6.7)
**Academic rank or position**		
	Instructor or tutor	3 (10)
	Lecturer	5 (16.7)
	Assistant professor	11 (36.7)
	Associate professor	7 (23.3)
	Professor	4 (13.3)
**Years of teaching experience**		
	0-5	1 (3.3)
	6-10	1 (3.3)
	11-15	13 (43.3)
	≥16	15 (50)
**Teaching field^a^ (multiple response)**		
	Gross anatomy	27 (90)
	Histology or cell biology or cytology	23 (76.7)
	Embryology or developmental biology	22 (73.3)
	Neuroanatomy or neurobiology	22 (73.3)
	Anatomy and physiology	15 (50)

^a^Multiple responses allowed; percentages for teaching fields use 30 as the denominator and therefore do not sum to 100%.

### Faculty Proficiency With Educational Technology

Overall, 73.3% (22/30) of respondents expressed confidence in their technological skills (N=30; mean 3.73, SD 1.01; median 4.0, IQR 3.25-4.00; 95% CI 3.35-4.11), with 56.7% (17/30) agreeing and 16.7% (5/30) strongly agreeing that they were proficient in using educational technologies (Figure S1 and Table S1 in Multimedia Appendix 3). A smaller proportion reported neutral views (5/30, 16.7%), while only 6.7% (2/30) disagreed and 3.3% (1/30) strongly disagreed. Shapiro-Wilk tests confirmed nonnormal distributions for all variables (all *P*<.05). Exploratory Kruskal-Wallis comparisons revealed no detectable differences in proficiency levels across academic ranks or age groups (all *P*>.05). Given small subgroup sizes (n=2-11 per category), these null findings are exploratory and should not be treated as evidence of true group equivalence*.* These findings indicate a generally high level of digital competence among United Arab Emirates anatomy educators, providing a favorable foundation for adopting AI-enhanced teaching tools and digital learning innovations.

### GenAI Use in Anatomy Education

Table 2 shows that most faculty used GenAI in teaching (28/30, 93.3%), led by ChatGPT (26/30, 86.7%), with smaller uptake of Gemini (11/30, 36.7%) and Bing Copilot (10/30, 33.3%). Fewer reported writing assistants (8/30, 26.7%), image generators (6/30, 20%), custom GPTs or plugins (5/30, 16.7%), or Claude (3/30, 10%); 6 of 30 (20%) listed other tools, and 2 of 30 (6.7%) reported no GenAI use. Use was typically weekly or daily, mainly for quiz generation, content summarization, and drafting teaching materials. Furthermore, survey responses to “In what ways have you used GenAI tools in your work as an anatomy educator?” in Table 3 indicated that use was concentrated in teaching content development, particularly generating quiz or examination questions (19/30, 63.3%), summarizing complex topics for lectures (16/30, 53.3%), and drafting instructional materials such as slides or handouts (15/30, 50%). Nearly half also reported research writing or manuscript support (13/30, 43.3%). Assessment-related uses were less frequent, including preparing student feedback or reflections (8/30, 26.7%) and grading assistance or rubric design (8/30, 26.7%), while visual content generation was reported by 7 of 30 (23.3%). A minority had not used GenAI yet (3/30, 10%).

**Table 2:** Self-reported uses of generative artificial intelligence (GenAI) tools among participating United Arab Emirates anatomy faculty (N=30)^a^.

GenAI tool used	Faculty, n (%)
ChatGPT (OpenAI)	26 (86.7)
Google Gemini (formerly Bard)	11 (36.7)
Bing Copilot (Microsoft or OpenAI)	10 (33.3)
Notion AI^b^ or Grammarly or writing assistants	8 (26.7)
DALL·E or Midjourney or image generators	6 (20)
Custom GPTs or plugins	5 (16.7)
Claude (Anthropic)	3 (10)
None	2 (6.7)

^a^GenAI tool platforms personally used for teaching. The faculty column shows the number of respondents selecting each platform; percentages are calculated out of 30. Multiple selections were permitted, so totals may exceed 100%.

^b^AI: artificial intelligence.

**Table 3:** Self-reported purposes for using generative artificial intelligence (GenAI) tools among participating United Arab Emirates anatomy faculty (N=30).

GenAI tool use	Faculty, n (%)
Generating quiz or examination questions	19 (63.3)
Summarizing complex topics for lectures	16 (53.3)
Drafting instructional materials	15 (50)
Research writing or manuscript support	13 (43.3)
Preparing student feedback or reflections	8 (26.7)
Grading assistance or rubric design	8 (26.7)
Generating anatomy images or diagrams	7 (23.3)
I have not used GenAI tools yet	2 (6.7)

### Attitudes Toward AI in Medical Education

**Figure 1:**
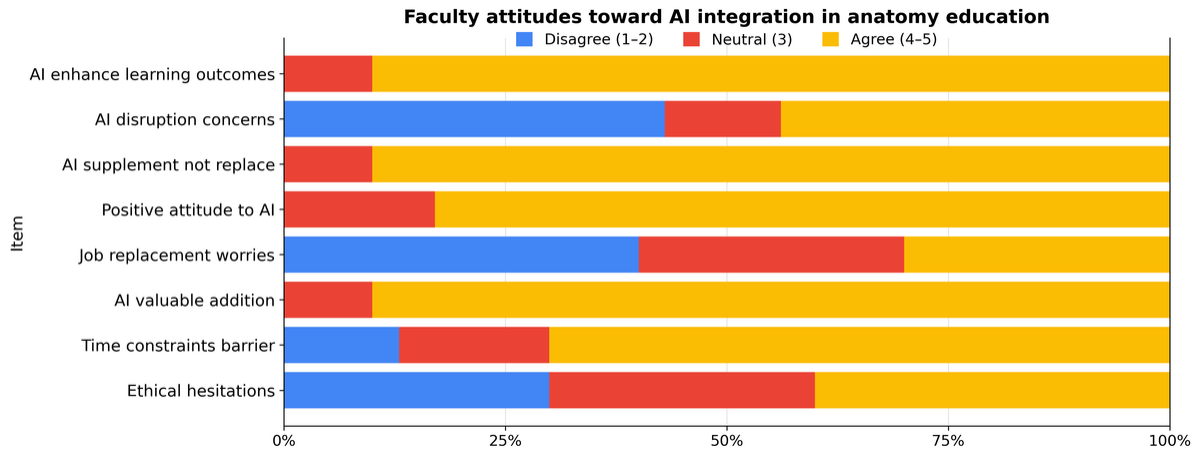
Attitude response patterns toward AI in anatomy education. Response patterns across 8 AI attitude domains showing percentage distribution of agreement levels. Blue bars represent disagreement (scores 1-2; disagree+strongly disagree), reflecting reservations; red bars represent neutrality (score 3), illustrating ambivalent or undecided attitudes; and yellow bars represent agreement (scores 4-5; agree+strongly agree), representing positive attitudes. All items demonstrated significant departures from normality (Shapiro-Wilk W 0.710-0.840; all *P*<.001). Exploratory group comparisons (Kruskal-Wallis H; academic rank: H=10.0-12.6; *P*=.01-.04; age group: H=8.8-10.0; *P*=.02-.03) revealed preliminary patterns, suggesting that assistant professors endorsed AI somewhat more highly than other ranks, and younger faculty reported greater time constraints. However, these differences are exploratory, given small subgroup sizes (n=2-18 per category), and should be interpreted with caution. The substantive finding is more noteworthy: across all demographic subgroups, faculty maintained predominantly positive attitudes (mean 3.60, SD 1.07; mean 4.5, SD 0.57; median 3.69, IQR 3.50-4.00), with internal consistency high (Cronbach α 0.7-0.9; McDonald ω 0.8), indicating broad consensus on AI’s educational role. The observed variation appears to reflect gradations of enthusiasm rather than fundamental disagreement. Power and precision limitations due to small subgroup sizes mean that these exploratory comparisons should be confirmed in larger, representative samples before treating them as definitive group differences (N=30). AI: artificial intelligence.

Figure 1 presents overall positive faculty attitudes toward integrating AI into anatomy education (mean 4.23, SD 0.73). Overall, the majority of respondents (83.3%) agreed or strongly agreed that AI can enhance learning outcomes (mean 4.30, SD 0.65) and represents a valuable addition to anatomy teaching (mean 4.33, SD 0.84). Nearly all participants endorsed the view that AI should supplement rather than replace human educators (mean 4.50, SD 0.57), reflecting a balanced approach to innovation. While ethical hesitations (30%; mean 2.97, SD 0.96) and job replacement worries (26.7%; mean 2.87, SD 1.04) persisted among some faculty, most respondents remained positive about AI’s pedagogical role. Time constraints (63.3%; mean 3.60, SD 1.07) and perceived disruption to existing teaching routines (30%; mean 3.20, SD 1.22) emerged as moderate barriers to adoption. These findings indicate that United Arab Emirates anatomy educators exhibit strong acceptance of AI’s educational potential, tempered by reasonable caution regarding its ethical and professional implications, an attitudinal profile consistent with early-stage adoption models.

### Barriers and Enablers to AI Integration in Anatomy Education

As shown in Table 4, the most prominent obstacles were capacity-related, notably time constraints, given the workload (mean 3.27, SD 1.17; 46.7% agreement) and training gaps (mean 3.13, SD 1.22; 36.7%), both rated as moderate barriers. Over a quarter of respondents (26.7%-30%) disagreed or strongly disagreed, highlighting variability in readiness across institutions. Budget or resource limitations (mean 2.63, SD 1.19; 53.3% disagreement) and academic-integrity concerns (mean 2.80, SD 1.10; 40% disagreement) were the least endorsed, indicating minor barriers overall. Conversely, motivation by student interest (mean 4.23, SD 0.86; 80% agreement) and institutional encouragement (mean 4.00, SD 1.14; 73.3%) emerged as strong facilitators, with very few dissenting responses (≤10%). Facilitators outweighed barriers, and the main obstacles were capacity-related (time or workload and training) rather than resource-related (budget), indicating that targeted professional development and workload support could capitalize on existing institutional encouragement and student-driven motivation to enable AI integration.

**Table 4:** Perceived barriers and facilitators to artificial intelligence integration in anatomy education among United Arab Emirates faculty^a^.

Factor type and domain			Item		Mean (SD)		Median (IQR)		Agreement (%)		Disagreement (%)		Severity
**Barriers**													
	Capacity constraints	Time or workload constraints		3.27 (1.17)		3.0 (3.0-4.0)		50		23.3		Moderate	
	Capacity constraints	Training gaps		3.13 (1.22)		3.0 (2.0-4.0)		46.7		30		Moderate	
	Quality concerns	Academic integrity		2.80 (1.09)		3.0 (2.0-4.0)		30		40		Minor	
	Resource constraints	Budget or resource limitations		2.63 (1.19)		2.0 (2.0-3.5)		26.7		53.3		Minor	
**Facilitators**													
	Extrinsic motivation	Motivation by student interest		4.23 (0.86)		4.0 (4.0-5.0)		80		3.3		Strong	
	Institutional support	Institutional encouragement		4.00 (1.15)		4.0 (4.0-5.0)		73.3		10		Strong	

^a^Responses were rated on a 5-point Likert scale (1=strongly disagree, 5=strongly agree). Agreement (%)=percentage scoring 4-5; agree+strongly agree; Disagreement (%)=percentage scoring 1-2; disagree+strongly disagree; severity classifies barriers (minor: mean<3.0; moderate: mean≥3.0) and facilitators (moderate: mean<4.0; strong: mean≥4.0). Capacity constraints (time and training) emerged as primary barriers, while social and institutional support provided strong facilitation. All items nonnormally distributed (Shapiro-Wilk *P*<.05). Exploratory comparisons revealed no detectable differences by academic rank or age (Kruskal-Wallis *P*>.05) in perceived barriers or enablers. However, this null finding should not be interpreted as evidence of true group equivalence; the small sample size and subgroup heterogeneity limit statistical power to detect meaningful differences if they exist. A larger, stratified sample would be needed to definitively test whether barriers differ by seniority, age, or experience. Descriptively, all faculty emphasized time and training as primary obstacles regardless of demographic background (N=30).

### Thematic SWOT Analysis

#### Overview

In the thematic SWOT analysis, coded segments were organized within a strengths-weaknesses-opportunities-threats structure, with weaknesses expressed more prominently and accompanied by recurrent support-needs statements. Themes clustered around infrastructure or resource readiness and training or professional development as core prerequisites for adoption, alongside concerns relating to time or workload, cost, ethics or privacy or integrity, and leadership or policy or governance. Smaller but salient strands captured pedagogical opportunities (including 3D personalization), caution about overreliance and erosion of critical thinking, and equity or access considerations. These themes were summarized within the SWOT framework (Textbox 1) and mapped to UTAUT2 constructs to contextualize faculty-perceived enablers and barriers to AI integration in anatomy education.

**Strengths, weaknesses, opportunities, and threats (SWOT) analysis of faculty perspectives on artificial intelligence (AI) integration in anatomy education, mapped to key constructs of the Unified Theory of Acceptance and Use of Technology 2 (UTAUT2).**

**Strengths (faculty or institutional strengths)**

- Supportive institutional climate (social influence and facilitating conditions that foster behavioral intention to adopt AI tools)
- Robust digital infrastructure (facilitating conditions that underpin the strong performance expectancy)
- Emerging professional development and skill building (facilitating conditions+effort expectancy)
- Innovation culture and growth mindset (reduces perceived effort expectancy and supports hedonic motivation+behavioral intention)

**Weaknesses (barriers and internal constraints)**

- Insufficient training and awareness (reduced effort expectancy and limited facilitating conditions)
- Time and workload burden (reduced effort expectancy and limited facilitating conditions)
- Infrastructure and IT support gaps (reduced performance expectancy, doubt AI will work reliably at the point of need)
- Policy and governance ambiguity (undermine social influence and behavioral intention—policy ambiguity can make faculty reluctant to fully embrace AI if they are not given clear rules on how to use it)
- Student preparedness and time pressures (without strengthened facilitating conditions, effort expectancy, and governance, AI adoption risks remaining fragmented and superficial)

**Opportunities (perceived benefits or future potential)**

- Enhanced anatomy learning outcomes (high performance expectancy; AI can make anatomy more comprehensible and memorable)
- Workload efficiency and scalability (improving both performance expectancy and effort expectancy reduces preparation and workflow burden)
- Personalization and engagement in anatomy pedagogy (performance expectancy [better learning] and hedonic motivation [more engaging, enjoyable experiences]).

**Threats (perceived risks and external pressures)**

- AI overreliance and deskilling (unhelpful habit formation and diminished performance expectancy for deeper, independent learning)
- Ethical and integrity risks (undermine social influence and behavioral intention)
- Loss of traditional, hands-on anatomy teaching (diminished performance expectancy, hedonic motivation threat to learning outcomes, and valued experiential and relational aspects of anatomy education)

Note: Open-ended responses (N=145 coded segments) were analyzed using Braun and Clarke’s reflexive thematic analysis and organized into 4 SWOT domains. Each quadrant with themes reflects patterned meaning across faculty narratives within the SWOT domains, illustrating how perceived strengths and opportunities (enablers) coexist with governance, ethical, and infrastructural vulnerabilities (weaknesses and threats) in AI-driven anatomy education. Each quadrant represents inductively derived subthemes linked deductively to UTAUT2 constructs: performance expectancy (perceived usefulness and teaching enhancement), effort expectancy (perceived ease of AI adoption), social influence (peer and student encouragement), facilitating conditions (institutional and technical supports), hedonic motivation (enjoyment and engagement), habit, and behavioral intention.

#### Current Strengths (Internal Assets)

Thematic analysis of the open-ended responses identified 4 interrelated themes reflecting strong internal capacities for AI integration in anatomy education (Table S1 in Multimedia Appendix 4). Collectively, these strengths map primarily onto the UTAUT2 constructs of facilitating conditions, performance expectancy, social influence, and hedonic motivation. Respondents described a generally supportive environment characterized by digital readiness, leadership encouragement, and a culture of experimentation.

Many educators credited their institutions with explicit endorsement of AI-enhanced teaching, describing “leadership promoting AI” and “supportive administrative, leadership drive toward technology adoption.” Such “strong support” signals organizational commitment and establishes a positive normative climate for faculty adoption. Faculty perceive encouragement from both senior management and departmental peers as lowering the perceived risk of innovation, thereby strengthening behavioral intention to engage with AI tools. Typical remarks included:

supportive administrative, leadership drive towards AI-driven pedagogy and incorporation into everyday work

AF30

my institution is very supportive

AF3

AI policies in place

AF14

A recurrent strength was the presence of solid digital infrastructure, “access to digital infrastructure such as high-speed internet, modern computers, and interactive displays” and “Internet infrastructure.” These assets provide essential technical foundations for integrating AI into anatomy teaching workflows. Respondents viewed the availability of virtual reality, simulation platforms, and medical holodecks as tangible evidence that AI integration is practical and sustainable. Representative statements include:

I use VR headsets and Medical Holodeck

AF4

we have high-speed internet, modern computers, and interactive displays

AF17

have immersive rooms, AI-backed simulators

AF11

The infrastructure (is) provided by the institutional Design Lab supports trying AI.

AF5

Several participants highlighted ongoing or planned professional development and skill-building initiatives: “faculty development sessions,” “certificate courses on AI in education,” and “formal training in integration.” Such structured learning opportunities reduce the cognitive and technical burden associated with AI use, reinforcing confidence and ease of use. Faculty see these opportunities as a bridge from conceptual awareness to confident practice. Examples of supporting statements include:

There are faculty development session and even certificate courses on AI in education

AF28

general AI courses offered to all.

AF20

Multiple responses emphasized an innovative culture and growth mindset as depicted by positive, experimental attitude, “readiness to explore and experiment with the available tools,” “curiosity to integrate AI,” and “general willingness to learn.” This intrinsic motivation reflects institutional cultures that reward innovation and experimentation. Enjoyment and curiosity act as important motivational drivers that complement the extrinsic facilitators of infrastructure and policy: “explore and experiment with available tools”(AF19) and “institutional encouragement” for technological integration (AF8).

#### Weaknesses (Internal Challenges)

The 61 coded weakness segments described a landscape of readiness gaps and resource strain that constrain otherwise positive attitudes toward AI in anatomy education (Table S2 in Multimedia Appendix 4). Inductively, 4 main themes emerged, which were then interpreted through the UTAUT2 lens.

The most widespread concern was the need for faculty upskilling for AI use and awareness gaps thereof, expressed in statements such as:

Many colleagues don’t know how to use AI tools.

AF21

Lack of training or awareness,

AF10

insufficient professional development in AI,

AF3

requests for formal training in the integration of AI-related technologies.

AF25

These findings highlight systemic and capacity bottlenecks that constrain AI adoption*.* The majority of participants wanted “increased awareness of the available AI resources” (AF13) and “formal training and collaborative research (evidence-based) opportunities underlining a pressing need for institutional investment in capacity building” (AF7).

Second, respondents described time, workload, and resourcing constraints as key internal barriers. Several references to high existing workload and calls for “protected time to experiment” and “less workload and better financial support” illustrate that frontline faculty often lack the time, hardware, and licenses needed to experiment, redesign sessions, and troubleshoot. These constraints weaken facilitating conditions and lower performance expectancy (doubts that AI will work reliably in real teaching situations) and further depress effort expectancy. These were expressed through statements such as:

need time and workload relief, protected space to experiment with tools, and improved hardware and connectivity

AF21

no access to time to develop skill

AF11

not enough time to learn or implement AI tools due to workload.

AF15

Third, ethical anxieties featured prominently across responses. Participants anticipated “risk of cheating and plagiarism” and “inappropriate reliance on generative outputs,” expressing fear that AI might compromise academic integrity if inadequately regulated. Such concerns influence social norms around AI use and erode behavioral intention, as educators hesitate to integrate tools that could foster academic misconduct or blur authorship accountability.

risk of cheating and plagiarism.

Concerns about academic integrity

Limited student preparedness

AF1

cadaver, xrays and patient case data will be uploaded to AI

AI holds promise for anatomy, but its success depends on ethical use, proper training, and ensuring it complements, not replaces, human instruction.

AF7

A final prominent theme was fragility or shortage of reliable AI infrastructure and information technology support: “Sometimes the network and lab equipment are not dependable,” “... limited AI enabled infrastructure or IT support,” one respondent wrote, while another summarized simply, “lack of institutional AI policy.” Even where digital resources existed, several educators worried about their uneven availability and sustainability across programs. The absence of clear institutional policies leaves uncertainty about acceptable practices, further weakening facilitating conditions and normative guidance. In contrast to the strong internal assets identified among strengths, weaknesses expose systemic shortfalls in readiness, particularly around faculty capability, protected time, and policy coherence.

#### Opportunities (External Enablers)

Thematic analysis of the opportunity statements revealed a forward-looking optimism toward AI’s potential to transform anatomy education (Table S3 in Multimedia Appendix 4). These opportunities align closely with the UTAUT2 constructs of performance expectancy, effort expectancy, hedonic motivation, and facilitating conditions. The current participants envisioned equity and concrete pedagogical and operational gains, with gains including cost, time, and workload.

A dominant theme in the Opportunities responses was the view that AI can strengthen conceptual understanding by making complex 3D anatomy more vivid, supporting personalized learning pathways, and enabling rapid formative feedback. Some participants explicitly anticipated that:

AI in anatomy education offers personalized learning, interactive 3D visualization, instant feedback, and efficient assessment,

AF5

AI can help generate diagrams and 3D models for better understanding.

AF18

be used as a tool that complements the available teaching resources since it also has its own limitations, particularly for practical courses like anatomy.

AF8

Furthermore, efficiency and productivity gains featured with statements such as “less time to prepare for lectures,” “efficient workflow with large groups,” and “streamlining work.” Others expected similar benefits through “long-term protection of time for faculty” and “AI will lessen workload,” as routine content generation and assessment tasks will be automated. Several noted potentials for “enhancing student engagement,” improved access for diverse and multilingual learners if AI was to be integrated thoughtfully as represented by:

“AI could make learning available to more students” in “different languages, forms and catering for different abilities.”

AF9

Students may be able to grasp certain concepts better with AI enhanced tools or visualization

AF14

AI in anatomy education offers personalized learning, interactive 3D models, and adaptive quizzes.

AF5

will provide more means to support students’ engagement and curiosity.

AF17

High performance expectancy and hedonic motivation dominate this quadrant, as educators see AI as a means to make anatomy learning more efficient, engaging, and accessible.

#### Threats (External Challenges)

The external risks and cautionary perspectives were also evident and were centered around educational quality and system reliability (open-ended threat responses; Table S4 in Multimedia Appendix 4). Although participants generally support AI integration, they cautioned against unintended consequences that could compromise learning depth, authenticity, and human engagement. These perceived risks align with the UTAUT2 constructs of habit, performance expectancy, social influence, and behavioral intention, reflecting a nuanced tension between enthusiasm for innovation and anxiety about its potential excesses.

The anatomy educators worried about potential overreliance on AI and erosion of critical thinking or clinical reasoning and practical skill development, warning that:

Over relying on AI tools may decrease students/educators ability to reasoning, imagination and self development.

AF4

Others noted:

Overreliance on AI could weaken hands-on skills

AF13

Decrease in students learning capacity and long-term memory, lack of hands-on training, lack of live social communication

AF23

unrealistic representation of anatomy

AF20

complete loss of traditional way of learning anatomy.

AF30

hard to know if assignments are genuine

AF11

AI in anatomy education may pose risks like data privacy issues, biased content, reduced human interaction, and high implementation costs.

AF6

Others flagged technical failures and infrastructural vulnerability, recognizing that system failures or licensing problems could disrupt classes: “If the system fails, teaching will be disrupted” (AF13). One participant remarked on the “lack of infrastructure, even pro-versions of the AI tools” (AF10), underscoring that the cost of subscriptions to commercially available teaching tools is currently being shouldered largely by anatomy educators. Plagiarism and academic integrity concerns were also raised repeatedly, reflecting anxiety about unmonitored AI use in assessments and scholarly work. More broadly, respondents noted that the growing integration of GenAI in anatomy education introduces additional threats, including data privacy risks, biased outputs, high implementation costs, and increasing difficulty in verifying the authenticity of students’ work. Finally, a subset of participants report “None” or “Nothing I can think of, if used wisely,” indicating conditional trust; AI is seen as safe if guided by thoughtful policies and pedagogy. Overall, threats describe a tension between the allure of innovation and a desire to protect core educational values, standards, and skills.

### Quantitative-Qualitative Integration Summary

The qualitative SWOT findings complemented the survey data by contextualizing the statistically dominant barriers and facilitators identified in Table 4. Quantitative ratings of time constraints due to existing workload and training deficit (moderate barriers) were elaborated through rich descriptions of heavy workload and capacity challenges, confirming limited facilitating conditions under the UTAUT2 framework. Likewise, the strong quantitative endorsement of motivation by student interest and institutional encouragement as facilitators was echoed in the SWOT opportunities, where learner enthusiasm and organizational innovation as well as supportive culture emerged as primary drivers of AI adoption. The synthesis, thus, revealed a coherent pattern of high perceived usefulness and social motivation counterbalanced by systemic limitations imposed by governance deficits, infrastructural limitations, and workload. Collectively, these results depict United Arab Emirates anatomy educators as being in a transitional phase of AI acceptance, optimistic about pedagogical benefits, yet constrained by governance, training, and ethical infrastructure gaps that must be addressed for sustainable, responsible implementation.

## Discussion

### Principal Findings

This study provides one of the first systematic accounts of United Arab Emirates anatomy educators’ patterns of use and perspectives on AI, showing strong but cautious uptake of generative tools in everyday work. Most faculty expressed confidence in their educational technological skills and had already experimented with GenAI, mainly using ChatGPT and similar systems for content creation, assessment support, and research-related tasks, with smaller but notable use for visualization and workflow streamlining. Attitudes toward AI in anatomy education were broadly positive and oriented toward augmentation rather than replacement of traditional teaching and assessment, with no significant attitudinal differences by rank, age, or experience. These patterns merit confirmation in larger cohorts, given this modest (N=30), conference-recruited sample’s power limitations for subgroup analysis (n=1-18). Quantitative barriers to AI use centered on time or workload pressures and training gaps, while student interest and institutional support or encouragement emerged as strong enablers, a pattern reinforced by SWOT themes of digital readiness, innovation or supportive culture, and concurrent concerns about ethics, governance, and deskilling. Interpreted through UTAUT2, these findings reflect high performance expectancy and supportive social influence but reduced effort expectancy under workload and training constraints, which together temper behavioral intention and keep AI use largely exploratory rather than routine. In this mixed methods interpretation, SWOT themes were mapped onto UTAUT2 constructs to illustrate how perceived benefits, barriers, and institutional supports cohere into an adoption profile for United Arab Emirates anatomy educators; however, this mapping is interpretive and should be understood as a heuristic rather than a definitive test of the theory.

### Faculty Readiness and Professional Development

Most respondents were already using GenAI tools daily or weekly for quizzes, lectures, and content preparation and reported a strong interest in AI’s educational value, yet only moderate confidence in their own skills. SWOT comments such as “many colleagues do not know how to use AI tools,” “lack of training or awareness,” and calls for “formal training in the integration of AI-related technologies” highlighted limited hands-on experience as a key weakness that constrains confident classroom use. In UTAUT2 terms, this combination of high perceived benefit and low practical confidence reflects reduced effort expectancy: faculty see AI as pedagogically useful (high performance expectancy) but technically demanding to learn and integrate, which dampens behavioral intention despite strong student interest and institutional encouragement. These findings point to the need for structured, workflow-aligned faculty development focused on concrete use cases, prompt design, and critical appraisal of AI outputs to convert enthusiasm into routine, pedagogically grounded integration in anatomy teaching. Similar readiness gaps are reported internationally, where educators endorse AI’s potential but describe low competence and limited time for meaningful adoption [9,11,39-41]. Regional work from the Middle East and North Africa and South East Asia similarly describes faculty who recognize AI’s potential, yet lack skills and call for structured capacity building [32,34,42]. Together with these reports, the present findings support targeted, context-sensitive upskilling in practical AI use, verification, and ethics as a key strategy to overcome low effort expectancy and enable sustainable adoption [43-45].

### Institutional Climate: Support Present, Policy Lagging

Quantitative data showed strong institutional encouragement (mean 4.00, SD 1.14, 73.3% agree+strongly agree) and student interest (mean 4.23, SD 0.86, 80% agree+strongly agree) as leading facilitators, alongside 93.3% GenAI adoption primarily for anatomy content creation and research-related support. Within the UTAUT2 framework, this pattern suggests strong social influence from students and institutional leadership, alongside generally favorable facilitating conditions (eg, adequate digital infrastructure and/or a supportive culture). Collectively, these factors are likely to enhance performance expectancy and, in turn, strengthen behavioral intention to adopt AI [20]. However, SWOT responses revealed gaps in AI disclosure rules, cadaveric or patient image governance, and assessment integrity policies, leaving faculty uncertain about acceptable use for anatomy-specific materials. This policy ambiguity weakens facilitating conditions and generates risk-averse behavior, stalling progression from exploratory to routine use even in a supportive climate [19,46]. These tensions mirror regional deanery reports of pro-AI attitudes coexisting with “immature” formal frameworks [29,47] and align with anatomy-specific governance concerns around image provenance and technology-mediated assessments [13,36], underscoring the need for discipline-specific policy in the United Arab Emirates.

### Ethics, Integrity, and Data Governance

Although mean scores for ethical worry were moderate, open-ended responses repeatedly cited risks of plagiarism and overreliance, the need to verify and document AI outputs, and sensitivities around patient or cadaveric data. Faculty worried that students might use AI to shortcut assignments, undermining hands-on skill development, deep spatial reasoning, and professionalism, threats that are particularly acute in anatomy’s experiential, cadaver-based learning. In UTAUT2 terms, such uncertainties erode facilitating conditions, as a lack of clear governance and oversight make faculty cautious despite high performance expectancy for AI-supported visualization and feedback. These concerns are consistent with international scholarship calling for strong human oversight of AI in teaching and assessment and warning against uncritical automation [36]. Multisite qualitative work in Saudi Arabia, concordant with current findings, reports early enthusiasm tempered by uncertainty about trustworthiness, bias, and privacy, emphasizing the need for explicit use norms [48,49]. While AI-related policy appears to be lagging in the current United Arab Emirates study, medical schools are advised at the institutional level to implement governance frameworks that include explicit acceptable-use statements, disclosure requirements and/or audit trails, and staff development focused on bias detection and verification [50]. This aligns with scoping reviews that advocate for structured, ethically grounded implementation rather than ad-hoc experimentation. In the United Arab Emirates context, the absence of formal AI policies leaves AF reliant on personal judgment, which can inhibit broader adoption by increasing uncertainty and perceived risk. Clear institutional guidelines would strengthen facilitating conditions and support confident, ethical integration of AI tools in anatomy curricula.

### Workload and Pragmatic Enablers

SWOT responses identified time and workload pressure as prominent barriers, with faculty citing heavy teaching loads, few anatomy-specific AI training opportunities, and a lack of protected time to experiment with tools. While some respondents anticipated that AI might eventually reduce effort (eg, by automating quiz generation or lesson planning), they consistently noted that these benefits would follow a “long learning curve” and initial workload increase due to prompt development and careful verification of outputs. This “time-savings paradox,” upfront effort before efficiency gains, is echoed in the broader AI-in-education literature [9,51,52]*.* One participant stressed the need for “time and workload relief, protected space to experiment with tools,” illustrating that adoption depends on capacity and support, not just access. Mapped onto UTAUT2, these findings show that high workload and limited support reduce effort expectancy and weaken facilitating conditions, thereby suppressing behavioral intention, whereas structured experimentation, repeated exposure, and organizational reinforcement can eventually consolidate AI use into a habit [20,53]. Editorial and policy guidance similarly recommend sandboxing, prompt-craft workshops, and verification checklists before expecting durable time savings at scale [54].

### Strengths Unique to Anatomy

A distinctive contribution of this study is that faculty did not describe AI in generic “productivity” terms alone; rather, they articulated discipline-specific affordances that map directly onto core challenges in anatomy teaching. Respondents consistently highlighted the potential for enhanced visualization and spatial support, including automated labeling of anatomical structures, adaptive quizzing, and access to high-fidelity 3D models, capabilities they framed as particularly valuable for topics where learners must build robust 3D mental models. Importantly, faculty linked these affordances to learning-process benefits, noting that well-designed tools could support spatial reasoning and reduce extraneous cognitive load, especially when visualization, labeling, and assessment cues are integrated coherently. This anatomy-specific emphasis, visual-spatial scaffolding, structure identification, and 3D conceptualization represent a notable and somewhat unique pattern in our dataset relative to broader AI adoption discussions in medical education.

Equally salient in our findings was a discipline-grounded boundary condition: faculty were not “anti-AI,” but they were explicit that AI must complement, not displace, laboratory-based, tactile, and relational forms of learning that are central to anatomy’s epistemology and professional socialization. Participants cautioned against overreliance, potential erosion of hands-on competence, and diminished human interaction and situated learning, mirroring early evaluations and commentaries that position tools such as ChatGPT, simulations, and automated resources as adjuncts rather than substitutes for dissection or prosection and cadaveric context [55-57]. Recent syntheses likewise argue for explicit human oversight, verification practices, and alignment with learning-science principles (including cognitive load) to protect fidelity and assessment validity in anatomy [16,58,59]. Taken together, this study adds a nuanced, anatomy-specific account: educators recognize powerful visual-spatial advantages, but they also define clear pedagogical “red lines” around authenticity, embodiment, and professional formation.

On the basis of these discipline-specific insights, we recommend that United Arab Emirates and wider Gulf Cooperation Council institutions move beyond generic AI workshops and instead implement structured, anatomy-tailored faculty development, competency-aligned training in prompt design for anatomical content, bias or accuracy appraisal, and ethical verification workflows. In parallel, institutions should operationalize governance addressing AI disclosure, acceptable use of cadaveric and patient images, and safeguards for assessment validity. Finally, given the regional commonalities identified in this cohort, Gulf Cooperation Council–wide collaboration (shared faculty-development hubs, shared evaluation checklists, and interuniversity policy alignment) could accelerate safe innovation while supporting broader digital-transformation ambitions in higher education [44].

### Study Limitations

This study has several limitations. This census-style pragmatic sample (N=30) of United Arab Emirates anatomy educators recruited primarily at a national conference (May 2025) plus brief follow-up reflects practical constraints but limits representativeness and statistical power. Convenience sampling underpowers robust subgroup analyses (n=1-18 per category; ~20%-35% power for medium effects) and precludes regression of UTAUT2 pathways; null results should therefore be interpreted as lack of detectable difference, not evidence of equivalence. Self-report measures may be influenced by social desirability or early-adopter bias, given the high GenAI use reported, although SWOT comments also captured critical perspectives and governance concerns. No a priori power calculation was feasible; consequently, the emphasis is on descriptive quantitative patterns and qualitative depth in an understudied context. Future research should use larger, stratified samples, longitudinal designs, and intervention trials to test whether AI-supported anatomy teaching improves learning outcomes and preserves assessment validity as technologies and institutional policies evolve. Triangulation of ordinal data and reflexive thematic SWOT across United Arab Emirates institutions as well as a discipline-specific focus are marked strengths.

### Conclusions

In this national snapshot, United Arab Emirates anatomy educators reported broadly positive attitudes toward AI, oriented toward augmentation rather than replacement of established practices. Faculty perceptions converged across quantitative results and SWOT analysis: strong enablers like institutional encouragement and student interest outweighed barriers of time or workload, training gaps, and integrity concerns. Notably, 93.3% already used GenAI tools, primarily ChatGPT for anatomy content creation, visualization or 3D simulation, and assessment support. Educators perceived pedagogical value in 3D visualization and adaptive quizzing, while cautioning against overreliance eroding tactile dissection skills essential for spatial reasoning. Faculty-identified needs centered on development, governance or disclosure policies, and assessment safeguards to maintain integrity amid GenAI use.

## Data Availability

The datasets generated or analyzed during this study are available from the corresponding author on reasonable request.

## Appendix

Multimedia Appendix 1 Survey prompts and the full instrument.

Multimedia Appendix 2 Completed reporting checklists for this mixed methods study: CHERRIES (web-based survey reporting), STROBE (observational reporting), and SRQR (qualitative reporting).

Multimedia Appendix 3 United Arab Emirates anatomy faculty members’ self-rated technology proficiency, familiarity with artificial intelligence and generative artificial intelligence tools, and the self-reported frequency of generative artificial intelligence use for teaching and other academic purposes (eg, lesson planning, assessment item writing, research writing, and administrative tasks).

Multimedia Appendix 4 Strengths, weaknesses, opportunities, and threats data.

## Abbreviations

AF: anatomy faculty
AI: artificial intelligence
CHERRIES: Checklist for Reporting Results of Internet E-Surveys
GenAI: generative artificial intelligence
SRQR: Standards for Reporting Qualitative Research
STROBE: Strengthening the Reporting of Observational Studies in Epidemiology
SWOT: strengths, weaknesses, opportunities, and threats
UTAUT: Unified Theory of Acceptance and Use of Technology
UTAUT2: Unified Theory of Acceptance and Use of Technology 2
