# Supplementary material for: Faculty Perspectives on AI Integration in Anatomy Education in the United Arab Emirates: Cross-Sectional Survey
Source: JMIR Med Educ. 2026 Apr 21;12:e87418. doi: 10.2196/87418 (PMC13146239; doi:10.2196/87418)
Supplement: Multimedia Appendix 1 [file mededu_v12i1e87418_app1.docx]

**Survey Tool for Anatomy Educators – AI in Medical Education (UAE)**

Dear Participant,

Thank you for taking the time to participate in this survey. This study aims to explore the perspectives, experiences, and readiness of anatomy educators in the United Arab Emirates (UAE) regarding the integration of artificial intelligence (AI) into medical education. As AI technologies increasingly influence healthcare and education, understanding the insights of anatomy faculty is essential for guiding curriculum development, professional training, and institutional policies.

Your responses will remain confidential and will be used solely for research purposes. The survey should take approximately 10–15 minutes to complete. Your input is highly valuable and will contribute to shaping the future of anatomy teaching in the AI era.

By proceeding with this questionnaire, you consent to participate in this study.

Thank you for your contribution.

**Prince LM Zilundu**

**Section 1: Demographics**

1. Institution name: _______________________
2. Academic rank: Lecturer / Assistant Professor / Associate Professor / Professor / Clinical Tutor
3. Teaching field (select all that apply): Anatomy / Histology / Embryology / Neuroanatomy / Physiology / Other: _______
4. Years of teaching experience:
   - 0–5
   - 6–10
   - 11–15
   - 16+
5. Institution type: Government / Private / Semi-private
6. Have you received formal training in digital education or AI tools? Yes / No

**Section 2: Familiarity and Use of AI**

*(Adapted from Masters & Ellaway, 2021; Sallam, 2023)*

1. How familiar are you with the use of AI in medical education?
   - Very familiar / Somewhat familiar / Slightly familiar / Not familiar at all
2. Have you used any AI-based tools in your teaching of anatomy?
   - Yes – regularly / Occasionally / No – but I plan to / No – and I don’t plan to
3. (b) Have you personally used AI tools in your assessments eg setting exam questions? Yes – regularly / Occasionally / No – but I plan to / No – and I don’t plan to
4. Which AI applications have you used or are interested in using? *(Select all that apply)*
   - ChatGPT or other LLMs
   - Adaptive platforms for self-paced learning
   - AI-assisted assessment or grading
   - Virtual anatomy simulations or 3D tools
   - Automated feedback or analytics dashboards
   - Not yet used any
5. In your opinion, how useful is AI in enhancing medical education (anatomy)? Extremely useful / Somewhat useful / Neutral / Not useful
6. To what extent is your institution supportive of AI adoption in teaching?

- Very supportive / Somewhat supportive / Neutral / Unsupportive / Not sure

**Section 3: Attitudes and Perceptions of AI**

Rate your agreement with the following statements:
(Strongly Agree / Agree / Neutral / Disagree / Strongly Disagree)

1. AI tools can improve student learning outcomes in anatomy education.
2. I feel confident evaluating AI-generated content (e.g., explanations, assessments).
3. AI should supplement but not replace traditional anatomy instruction.
4. Ethical concerns about AI use (e.g., plagiarism, misinformation) affect my willingness to use it.
5. AI could help reduce workload for anatomy instructors.
6. AI may risk oversimplifying complex anatomical knowledge.
7. Students are ready to engage with AI-enhanced learning tools.
8. There is a clear institutional policy on responsible use of AI in teaching.

**Section 4: Barriers and Enablers**

1. What barriers do you perceive in adopting AI in anatomy education? *(Select all that apply)*

- Lack of institutional policy
- Lack of training or awareness
- Limited infrastructure or IT support
- Concerns about academic integrity
- Limited student preparedness
- Other (please give details)
- None / Not applicable

1. What would support you in adopting AI-enhanced teaching tools? *(Open-ended)*

**Section 5: Reflections and Experiences (Open ended)**

**Section 5: Reflections and Experiences (SWOT-Based Open-Ended)**

**A. Strengths**

1. In your view, what are the main strengths or advantages of integrating AI into anatomy education in your institution?
   - What successful experiences have you had using AI?
   - Can you share examples of improved teaching or student engagement due to AI?

**B. Weaknesses**
2. What weaknesses or challenges have you observed in using AI for anatomy instruction?

- What limitations or difficulties have you faced—technological, pedagogical, or otherwise?
- How have these issues affected your teaching or student outcomes?

**C. Opportunities**
3. What future opportunities do you see for using AI in anatomy education?

- Are there new areas of teaching, research, or student learning where AI could make a positive impact?
- How could AI contribute to advancing faculty development or curriculum innovation?

**D. Threats**
4. What potential risks or threats does AI integration pose for anatomy education?

- Do you have ethical, professional, or practical concerns about long-term AI adoption?
- How might these risks be minimized or managed at your institution?

**General Reflection and Follow-Up**
5. Do you agree that SWOT analysis is helpful for shaping AI policy and professional development in anatomy education? Why or why not?
6. Would you recommend more structured SWOT-based discussions in future faculty development workshops? Please elaborate.
