# Supplementary material for: Faculty Perspectives on AI Integration in Anatomy Education in the United Arab Emirates: Cross-Sectional Survey
Source: JMIR Med Educ. 2026 Apr 21;12:e87418. doi: 10.2196/87418 (PMC13146239; doi:10.2196/87418)
Supplement: Multimedia Appendix 3 [file mededu_v12i1e87418_app3.docx]

**Figure S1. Technology Proficiency and Artificial Intelligence Familiarity Among UAE Anatomy Faculty Members.**

*Educational technology proficiency distribution among 30 anatomy faculty members showing 73.3% with high proficiency (scores 4-5), 16.7% with moderate proficiency (score 3), and 10.0% with low proficiency (scores 1-2) (Mean ± SD = 3.73 ± 1.01; Median = 4.0). Shapiro-Wilk tests confirmed non-normal distributions for all variables (all p < 0.05). Kruskal-Wallis tests revealed no significant differences in proficiency levels across academic ranks or age groups (all p > 0.05). N = 30 for all analyses.*


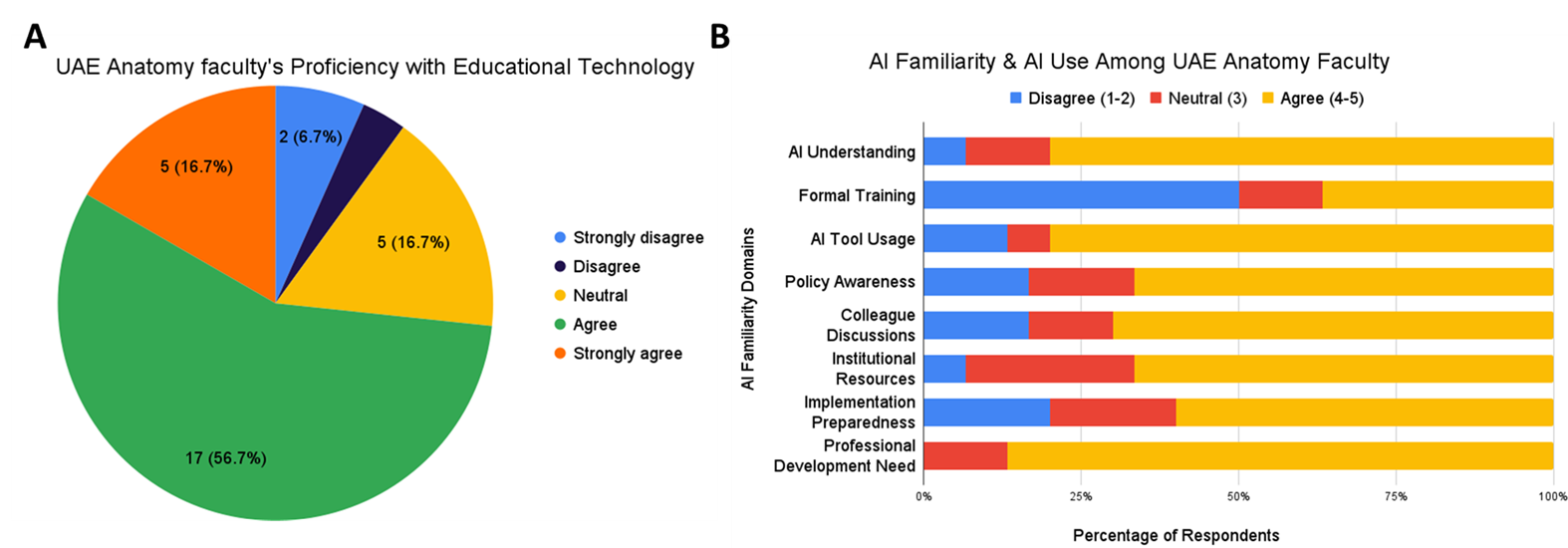


**Table S1.** Self-reported *frequency* of GenAI use for teaching or academic purposes (single response): daily, weekly, occasional (a few times per semester), or never. Bars show n respondents per category (percentages out of N = 30).

| **Frequency** | **Number of faculty, N** | **%** |
| --- | --- | --- |
| Daily | 12 | 40.0 |
| Weekly | 8 | 26.7 |
| Occasionally (a few times per semester) | 8 | 26.7 |
| Never | 2 | 6.7 |
