# Supplementary material for: Faculty Perspectives on AI Integration in Anatomy Education in the United Arab Emirates: Cross-Sectional Survey
Source: JMIR Med Educ. 2026 Apr 21;12:e87418. doi: 10.2196/87418 (PMC13146239; doi:10.2196/87418)
Supplement: Multimedia Appendix 4 [file mededu_v12i1e87418_app4.docx]

**Table S1.** Strengths codebook (faculty & institutional strengths supporting AI use).

| **Domain** | **Initial Code** | **Subtheme** | **Theme** | **Example Quote (verbatim)** | **UTAUT2 Link** | **Frequency (n)*** | **Representative Interpretation** |
| --- | --- | --- | --- | --- | --- | --- | --- |
| Strength | “Full support. My institution very strong on AI” | Institutional encouragement | Supportive institutional climate for AI adoption | “Full support. My institution very strong on AI” | **Social influence (SI); Facilitating conditions (FC); Behavioral intention (BI)** | ≈10 | Faculty perceive strong leadership encouragement and a pro-AI climate, which normalizes experimentation and future adoption. |
| Strength | “Leadership promoting AI” | Leadership advocacy | Strategic leadership for AI | “Leadership promoting AI” | **SI; FC** | ≈9 | Senior leadership are seen as active champions of AI, signalling institutional priority and reducing perceived risk of engagement. |
| Strength | “Access to digital infrastructure, such as high-speed internet, modern computers, and interactive displays.” | Infrastructure & tools | Robust digital and technical infrastructure | “Access to digital infrastructure, such as high-speed internet, modern computers, and interactive displays.” | **FC; Performance expectancy (PE)** | ≈9 | Existing infrastructure (internet, hardware, visualization labs, VR tools) is viewed as a solid foundation for AI-enhanced anatomy teaching. |
| Strength | “I use VR headsets and Medical Holodeck” | Existing advanced tools in use | Established technology-enhanced practice | “I use VR headsets and Medical Holodeck” | **PE; Habit** | ≈5 | Some faculty already integrate advanced tools (VR, simulations, immersive rooms), creating a habit base for extending into AI. |
| Strength | “There are faculty development sessions and even certificate courses… on AI in education.” | Training opportunities | Emerging AI-focused professional development | “There are faculty development sessions and even certificate courses being given by the university on AI in education.” | **FC; Effort expectancy (EE)** | ≈5 | Structured development opportunities lower perceived effort and build skills for meaningful AI adoption. |
| Strength | “Readiness to explore and experiment with the available tools” | Growth mindset | Positive innovation culture | “Readiness to explore and experiment with the available tools” | **Hedonic motivation (HM); BI** | ≈6 | Faculty describe curiosity and willingness to experiment, framing AI as an exciting space rather than a threat. |
| Strength | “Policies in place” | Existing governance | Early-stage AI governance structures | “Policies in place” | **FC; SI** | ≈3 | Where policies already exist, they are perceived as supportive guardrails rather than constraints. |

**Table S2.** Weaknesses codebook (barriers & internal constraints).

| **Domain** | **Initial Code** | **Subtheme** | **Theme** | **Example Quote** | **UTAUT2 Link** | **Frequency (n)** | **Representative Interpretation** |
| --- | --- | --- | --- | --- | --- | --- | --- |
| Weakness | “Lack of training or awareness” | Skill and knowledge gaps | Limited faculty readiness for AI | “Lack of training or awareness” | **Effort expectancy (EE); FC** | 16 | Many faculty feel underprepared to use AI tools, perceiving them as effortful and unfamiliar without structured upskilling. |
| Weakness | “Limited infrastructure or IT support” | Infrastructure constraints | Uneven facilitating conditions | “Limited infrastructure or IT support” | **FC; PE** | 17 | Inconsistent IT support and hardware gaps undermine confidence that AI tools will function reliably in real teaching contexts. |
| Weakness | “Lack of institutional policy” | Policy ambiguity | Perceived policy vacuum | “Lack of institutional policy” | **FC; SI** | 9 | Unclear regulations about AI use create uncertainty and hesitation, even where attitudes are positive. |
| Weakness | “Concerns about academic integrity” | Integrity concerns | Uncertainty about fairness and misuse | “Concerns about academic integrity” | **SI; BI** | 10 | Faculty worry about cheating, inappropriate AI assistance, and how to uphold standards, dampening intention to adopt AI in assessment. |
| Weakness | “Limited student preparedness” | Student digital/learning readiness | Unequal student readiness for AI-enhanced learning | “Limited student preparedness” | **PE; BI** | 9 | Perceived variation in students’ skills and maturity raises doubts about equitable benefits from AI tools. |
| Weakness | “Acceaa to time to develop skill” | Time/workload constraints | Competing demands on faculty time | “Access to time to develop skill” | **EE; FC** | ≈7 | Faculty emphasize limited time and heavy workloads as major obstacles to learning, testing, and integrating AI tools. |
| Weakness | “Less workload and better financial support” (as a wish) | Resource expectations | Under-resourced faculty role | “Less workload and better financial support” | **FC; PE** | ≈5 | Perceived lack of financial and workload support suggests that meaningful AI adoption requires resourcing beyond goodwill. |

**Table S3.** Opportunities codebook (perceived benefits & future potentials).

| **Domain** | **Initial Code** | **Subtheme** | **Theme** | **Example Quote** | **UTAUT2 Link** | **Frequency (n)** | **Representative Interpretation** |
| --- | --- | --- | --- | --- | --- | --- | --- |
| Opportunity | “Better 3D imagination… easy and quick learning” | Conceptual understanding & visualization | Enhanced learning outcomes | “For students: 1. Better 3D imagination 2. Easy and quick learning…” | **PE** | ≈15 | AI is expected to deepen anatomical understanding, particularly 3D spatial reasoning and concept clarity. |
| Opportunity | “Clear understanding of subject” / “knowledge retention, positive view of subjects” | Retention & attitudes | Improved retention and attitudes toward anatomy | “knowledge retention, positive view of subjects” | **PE; BI** | ≈8 | Faculty anticipate that AI tools will help students retain information and feel more positive about demanding content. |
| Opportunity | “Less time to prepare for lectures… If appropriately used can be of some use in teaching.” | Efficiency and workload reduction | Productivity gains for teaching | “Less time to prepare for lectures, questions and case studies.” | **PE; EE; FC** | ≈9 | AI is viewed as a way to streamline preparation, especially for cases, questions, and large-group sessions. |
| Opportunity | “Efficient workflow with large groups, enhancing student engagement” | Scaling & engagement | Scalable, engaging teaching at cohort level | “Efficient workflow with large groups, enhancing student engagement” | **PE; HM** | ≈7 | AI is seen as enabling more interactive, engaging learning even with large enrollment sizes. |
| Opportunity | “AI in anatomy education offers personalized learning, interactive 3D models…” | Personalization & interactivity | Tailored and immersive learning experiences | “AI in anatomy education offers personalized learning, interactive 3D models…” | **PE; HM** | ≈7 | Respondents anticipate more individualized, immersive experiences that respond to learner needs. |
| Opportunity | “remote learning (if trained) platform” | Access & flexibility | Expanded access and flexible delivery | “remote learning (if trained) platform” | **PE; SI** | ≈4 | AI-supported tools are perceived as extending anatomy learning beyond the physical classroom, particularly for remote or flexible options. |
| Opportunity | “Abreast in the recent trends and practices.” | Future-proofing | Staying current with evolving practice | “Abreast in the recent trends and practices.” | **PE; BI; HM** | ≈4 | Using AI is framed as necessary for keeping both students and curricula aligned with global trends. |

**Table S4.** Threats codebook (perceived risks & external pressures).

|  |  |  |  |  |  |  |  |
| --- | --- | --- | --- | --- | --- | --- | --- |
| **Domain** | **Initial Code** | **Subtheme** | **Theme** | **Example Quote** | **UTAUT2 Link** | **Frequency (n)** | **Representative Interpretation** |
| Threat | “Over relying on AI tools may decrease students/educators ability to reasoning, imagination and self development” | Overreliance & de-skilling | Erosion of core cognitive skills | “Over relying on AI tools may decrease students/educators ability to reasoning, imagination and self development” | **Habit; PE; BI** | ≈8 | Faculty worry that habitual AI use could weaken students’ independent reasoning and problem-solving abilities. |
| Threat | “Decrease in students learning capacity and long-term memory…” | Reduced learning depth | Superficial learning and poor retention | “Decrease in students learning capacity and long-term memory…” | **PE; BI** | ≈4 | Some fear that AI may encourage shallow learning and undermine long-term retention of anatomy. |
| Threat | “Data privacy issues… reduced human interaction, and high implementation costs.” | Ethics, privacy & cost | Ethical and implementation risks | “AI in anatomy education may pose risks like data privacy issues… reduced human interaction, and high implementation costs.” | **FC; SI; BI** | ≈5 | Concerns extend beyond pedagogy to privacy, cost, and the erosion of authentic teacher–student interaction. |
| Threat | “Plagiarism and technical difficulties” | Academic integrity & reliability | Misuse and technical fragility | “Plagiarism and technical difficulties” | **SI; FC; BI** | ≈5 | Risks of cheating, unreliable outputs, and breakdowns lower trust in AI-based assessments and resources. |
| Threat | “Complete loss of traditional way of learning anatomy” | Loss of traditional practice | Erosion of hands-on and specimen-based learning | “Complete loss of traditional way of learning anatomy” | **PE; HM; BI** | ≈7 | Respondents fear AI could displace dissection and specimen-based learning, which they see as irreplaceable. |
| Threat | “Complaceny… Lack of initiative to prepare or explore new material” | Complacency & dependency | Reduced teaching initiative | “Complaceny; Lack of initiative to prepare or explore new material” | **Habit; PE** | ≈4 | AI is seen as potentially discouraging active preparation and pedagogical creativity among both students and staff. |
| Threat | “I can not imagine any problem by now” / “Nothing I can think of, if used wisely” | Perceived absence of risk | Conditional trust in AI | “Nothing I can think of, if used wisely” | **PE; BI** | ≈8 | A minority express confidence that, under thoughtful use, AI poses no major threat, highlighting a more optimistic stance. |
|  |  |  |  |  |  |  |  |
